# Emerging agents and regimens for polycythemia vera and essential thrombocythemia

**DOI:** 10.1186/s40364-021-00298-5

**Published:** 2021-05-28

**Authors:** Rory M. Shallis, Nikolai A. Podoltsev

**Affiliations:** Section of Hematology, Department of Internal Medicine, Yale University School of Medicine and Yale Cancer Center, 333 Cedar Street, PO Box 208028, New Haven, CT 06520-8028 USA

**Keywords:** Essential thrombocythemia, ET, Newer, Novel, Polycythemia vera, PV

## Abstract

Polycythemia vera (PV) and essential thrombocythemia (ET) are both driven by JAK-STAT pathway activation and consequently much of the recent research efforts to improve the management and outcomes of patients with these neoplasms have centered around inhibition of this pathway. In addition to newer JAK inhibitors and improved interferons, promising novel agents exploiting a growing understanding of PV and ET pathogenesis and disease evolution mechanisms are being developed. These agents may modify the disease course in addition to cytoreduction. Histone deacetylase, MDM2 and telomerase inhibitors in patients with PV/ET have demonstrated clinically efficacy and serve as chief examples. Hepcidin mimetics, limiting iron availability to red blood cell precursors, offer an exciting alternative to therapeutic phlebotomy and have the potential to revolutionize management for patients with PV. Many of these newer agents are found to improve hematologic parameters and symptom burden, but their role in thrombotic risk reduction and disease progression control is currently unknown. The results of larger, randomized studies to confirm the early efficacy signals observed in phase 1/2 trials are eagerly awaited.

## Introduction

Polycythemia vera (PV) and essential thrombocythemia (ET) are the most common Philadelphia chromosome-negative myeloproliferative neoplasms (MPNs). This observation is not solely due to their incidence of 1.0–2.0 per 100,000 person-years, but also patients’ near-normal life expectancy, culminating in a prevalence of approximately 25–50 per 100,000 people [1]. Although biologically-distinct diseases, ET and PV share a pathogenesis generally rooted in JAK-STAT activation, which prompts the unregulated proliferation of hematopoietic stem/progenitor cells (HSCs). Ninety nine percent of PV is driven by acquired mutations in the Janus kinase 2 (*JAK2*) gene, almost always *JAK2*-V617F [2–4]. A *JAK2*-V617F mutation is found in approximately 50% of patients with ET [5–8]. In contrast to PV, about 20–25 and 5% of ET is characterized by a *CALR* and *MPL* mutation, respectively [9–12]. Up to 10% of ET has no detectable canonical driver mutation and is typically regarded as being “triple negative,” but rare non-canonical *CALR, MPL* and *JAK2* variants have been detected in “triple negative” disease [3, 13].

Although predicting a near-normal life expectancy, true to their categorization as myeloid malignancies, PV and ET are associated with an increased risk of the development of thrombosis, secondary myelofibrosis, myelodysplastic syndrome (MDS) and transformation to acute myeloid leukemia (AML) with these entities predicting a shorter life expectancy than otherwise expected with a PV or ET diagnosis itself [14–17]. In addition to these tangible outcomes, patients with PV and ET are also frequently burdened with symptoms including fatigue, night sweats, headaches, pruritus, early satiety/weight loss, and symptomatic splenomegaly [18]. It is for these reasons as well as to reduce the risk of arterial/venous thrombosis and bleeding that therapy for PV or ET is initiated. However, despite this need and a firm understanding of pathogenesis, hydroxyurea, anagrelide, pegylated interferon alfa 2a (PEG-IFN alfa-2a), ruxolitinib and busulfan constitute the bulk of cytoreductive therapies available to providers charged with the care of patients with PV and ET, but most have little to no evidence of disease-modifying activity. Inteferons have been used for the management of MPN for > 30 years and are favored by some providers due to their immunomodulatory, anti-inflammatory, antiangiogenic and anti-proliferative properties [19]. The introduction of pegylated interferon preparations improved tolerability, but are still associated with flu-like, autoimmune and psychiatric side effects [19]. A recent meta-analysis of 44 studies including 1359 patients treated with interferon (730 ET, 629 PV) showed high response rates among patients with PV (overall response rate [ORR] of 81%, complete hematologic response [CHR] of 59%) and ET (ORR of 77%, CHR of 49%) without significant differences observed between pegylated and non-pegylated formulations [20].

In addition to the high rates of CHR, symptomatic improvement and improvement in or resolution of need for therapeutic phlebotomy, many patients with PV and ET may experience a sustained decrease in *JAK2*-V617F allelic burden with a potential for disease modification due to malignant clone targeting by interferon [21]. A recent study evaluated interferon discontinuation among patients with PV and ET who accomplished CHR and demonstrated this practice to be safe with persistence of CHR particularly in patients with a driver mutation variant allele frequency < 10% at time of discontinuation; however disease transformation could not be evaluated due to the very low incidence of events [21]. Due to the overall favorable prognosis of patients with PV and ET, studies of survival and disease transformation may be challenging to conduct. The reduction of driver mutation allelic burden may be a feasible surrogate, but its relation to long-term outcomes is uncertain. Lifelong treatment represents a major burden for these chronically-ill patients and have to be well-tolerated without significant toxicities. Interferons may be especially attractive for the management of younger patients with PV or ET due to their lack of teratogenicity. The only unequivocal disease-modifying therapy is allogeneic hematopoietic stem cell transplantation (alloHCT) for which many patients with long life expectancy are not reasonable candidates.

Newer therapies with disease-modifying potential are needed for patients with PV and ET, particularly those patients deemed to have disease that is “higher risk” or resistant to currently available therapies. In this review we discuss some of the emerging and more promising therapies that are being explored and may soon become available to patients with PV or ET as well as some of the prospects for future investigation.

## Optimizing the current treatment options

### JAK inhibitors

Perhaps the greatest asset to the development of better or novel therapies for PV was the 2005 discovery of the gain-of-function *JAK2* V617F exon 14 mutation and the recognition that it drives 95% of PV cases; most of the remaining cases of PV are explained by the presence of a *JAK2* exon 12 mutation that was discovered 2 years later in 2007 [7, 22, 23]. The majority of contemporary therapies, including hydroxyurea, busulfan and interferons do not target the principal upstream element of JAK-STAT pathway activation. The JAK 1/2 tyrosine kinase inhibitor ruxolitinib is found to downregulate malignant MPN clone expression of pro-inflammatory cytokines like TNF-α and IL-6, which appear to be heavily dependent on JAK1 and JAK2-mediated activation of STAT3 [24–26]. In the RESPONSE trial, when compared with “standard therapy” (including hydroxyurea, interferon, anagrelide, lenalidomide/thalidomide, pipobroman, but excluding ^32^P, busulfan and chlorambucil), ruxolitinib demonstrated superior benefit with regards to hematocrit control, spleen volume reduction, and symptom burden reduction in patients intolerant of or with inadequate response to hydroxyurea [27]. Later follow-up and meta-analyses also supported its benefit in reducing the incidence of thrombosis compared to these standard therapies [28, 29]. Based on the RESPONSE trial results, ruxoltinib in 2014 became the first Food and Drug Administration (FDA)-approved therapy for PV, specifically for patients with PV who are resistant to or intolerant of hydroxyurea. The randomized, phase 3 RESPONSE-2 trial confirmed these findings in patients specifically *without* palpable splenomegaly [30]. Other JAK inhibitors, have been studied in PV. Gandotinib, a pan-JAK inhibitor with dose-dependent selectivity for JAK2, demonstrated a 95% ORR in patients with PV (including those previously receiving ruxolitinib) [31, 32] (Table 1).

**Table 1:** Summary of clinical data for emerging/novel therapies for polycythemia vera

Agent	Mechanism of action	Clinical data	Reference
Gandotinib	Pan-JAK inhibition	Phase 1 trial; *N* = 6 (median age 62.5 years) Results: 3 patients had clinic-hematologic partial response [31] Phase 2 trial; *N* = 20 Results: 4 patients (20%) had CR and 15 (75%) had PR, thus an ORR of 95% [32]	[31, 32]
Ruxolitinib + PEG-IFN alfa-2a	JAK1/2 inhibition + immunomodulation/antiangiogenesis	Phase 2 trial; *N* = 32 (median age 57 years, 66% high-risk, 94% with prior interferon exposure) Results: 10 patients (31%) had remission with 3 (9%) in CR and another 7 (22%) with PR. Symptom burden was improved with only 6% drop-out rate.	[33]
Ropeginterferon alfa-2b	Immunomodulation/antiangiogenesis	Phase 3 trial; *N* = 222 (median age 58–60 years) randomized to ropeginterferon alfa-2b Results: improved rates of composite CHR when compared with hydroxyurea (53% vs. 38%, *p* = 0.044) and improved symptom burden at 36 months follow-up	[34]
Givinostat	Histone deacetylase inhibition	Phase 1b/2 trial; *N* = 47 Results: ORR of 81%, but significant toxicity (35–50% grade ≤ 3 diarrhea, thrombocytopenia and creatinine elevation)	[35]
Idasanutlin	MDM2 inhibition	Phase 2 trial; *N* = 27 (median age 56 years, 26% with prior ruxolitinib exposure) Results: 9 patients (56%) achieved hematocrit control by 32 weeks of therapy, however significant gastrointestinal toxicity	[36]
PTG-300	Hepcidin mimesis	Phase 2 trial; *N* = 13, all requiring therapeutic phlebotomy (mean age 57.4 years, 46% high-risk) Interim data showing normalization of iron stores, 100% therapeutic phlebotomy-independence and improved symptom burden	[37]

*Abbreviations*: *CHR* complete hematological response, *CR* complete response, *ORR* overall response rate, *PEG-IFN alfa-2a* pegylated interferon alfa 2a, *PR* partial response

Given the understanding of the role of JAK-STAT pathway activation in ET, JAK inhibition has been studied in this MPN as well. The first study of ruxolitinib in ET evaluated 39 patients who were refractory to or intolerant of hydroxyurea reported a favorable safety profile and that a majority of patients experienced white blood cell count, platelet count, spleen size and symptom burden reductions [38]. The MAJIC-ET trial randomized 110 patients with ET who were refractory to or intolerant of hydroxyurea to either ruxolitinib or best available therapy with most patients in the latter arm receiving anagrelide [39]. No differences in hematologic responses, thrombosis/bleeding or disease progression were observed between arms; however, a greater and more rapid symptom burden reduction was noted for patients receiving ruxolitinib [39]. RESET 272 is a phase 2 trial currently evaluating ruxolitinib in comparison to anagrelide in patients with ET who were refractory to or intolerant of hydroxyurea (NCT03123588). RUXBETA (NCT02962388) and Ruxobeat (NCT02577926) are ongoing phase 3 trials in the same population comparing ruxolitinib with best available therapy (including anagrelide and interferons). With regards to other JAK inhibitors, the same studies that evaluated gandotinib in PV reported a 90% ORR in ET, including 44% of patients with ET and no detectable *JAK* mutation [31, 32] (Table 2).

**Table 2:** Summary of clinical data for emerging/novel therapies for essential thrombocythemia

Agent	Mechanism of action	Clinical data	Reference
Gandotinib	Pan-JAK inhibition	Phase 1 trial [31] *N* = 1 Results: single included patients with ET had a PR Phase 2 trial [32] *N* = 21 (median age 60 years) Results: 3 patients (14%) had CR, 16 (76%) had PR, thus ORR of 90%	[31, 32]
Imetelstat	Telomerase activity inhibition	Phase 2 trial *N* = 18 (44% age > 60 years, 44% with *JAK2* mutation; 94 and 72% with prior hydroxyurea and anagrelide exposure, respectively) Results: 100% hematologic response rate with 89% CHR rate	[40]

*Abbreviations*: *CHR* complete hematological response, *ORR* overall response rate

Limiting the enthusiasm for frontline ruxolitinib or other JAK inhibitor therapy for PV or ET is the lack of evidence for disease course modification including long-term risk of secondary (post-PV and post-ET myelofibrosis). Attempts to build upon the practical use of a JAK inhibitor for disease driven by JAK-STAT activation have included studies of combination therapy. The final results of a phase 2 trial of the combination of ruxolitinib with PEG-IFN alfa-2a showed the combination to be reasonably well-tolerated, to decrease both *JAK2* V617F allelic burden and symptoms as well as ultimately lead to remission in 10 of 32 patients (31%) with PV who were almost entirely previously-intolerant of or refractory to PEG-IFN alfa-2a [33]. Randomized studies are needed to confirm the independent benefit of the addition of ruxolitinib to interferons for this population of patients.

### Interferons

Inteferons remain a promising class of drugs with the potential for disease modification among patients with MPN [20]. Alternative interferon preparations for PV and ET patients are being studied and have largely been developed to improve upon the burdensome weekly administration schedule of currently-used pegylated interferons (e.g. PEG-IFN alfa-2a). Ropeginterferon alfa-2b is produced by *Escherichia coli* transformed with a human IFN plasmid and specifically modified by an additional N-terminus proline residue and single isomer that leads to an extended half-life allowing for dosing every 2 weeks [41, 42]. The PROUD-PV and CONTINUATION-PV phase 3 trials randomized 257 patients with PV and ≤ 3 years of cytoreductive therapy exposure 1:1 to receive ropeginterferon alfa-2b every 2 weeks or hydroxyurea [34, 41]. While PROUD-PV demonstrated no clear difference between treatment arms, patients treated with ropeginterferon alfa-2b on CONTINUATION-PV had better rate of composite complete hematological response with reduced symptom burden when compared with hydroxyurea (53% vs. 38%, *p* = 0.044) at 36 months follow-up [34]. In addition, the molecular response, defined as reductions in *JAK2*-V617F allelic burden, was significantly improved in the ropeginterferon alfa-2b arm when compared with standard therapy (66% vs. 27%; *p* < 0.0001); these results are durable based on 5 years of follow-up [43]. These data demonstrated that ropeginterferon alfa-2b was at least non-inferior to hydroxyurea for PV patients and led to the European Medical Agency approval of ropeginterferon alfa-2b for the frontline treatment of polycythemia without significant splenomegaly in 2019; the FDA has accepted a Biologics License Application for ropeginterferon alfa-2b for this indication within the United States and a decision is expected during 2021.

Interferons are also among the cytoreductive options for patients with ET. Similar to endeavors dedicated to PV, ropeginterferon alfa-2b is being currently studied in a phase 3 trial randomizing patients who are refractory to or intolerant of hydroxyurea to receive either anagrelide or ropeginterferon alfa-2b (NCT04285086).

### Novel agents

In addition to efforts to optimize the use of current standard of care classes of agents, several novel compounds are being studied as therapy for PV. Targeting of epigenetic pathways, particularly the varied status of post-translational histone acetylation/de-acetylation which contribute to the regulation of gene transcription critical to cell cycling, offer a promising therapeautic opportunity [44]. The histone deacetylase inhibitors (HDACis) are theorized to reverse histone/DNA complex compaction and thus open the chromatin structure to restore pro-apoptotic gene transcription [44]. Although an oversimplified view, the HDACis are hypothesized to have a role in the treatment of MPN [45]. The HDACis vorinostat, panobinostat and givinostat have demonstrated that ability to inhibit proliferation and induce apoptosis of *JAK2*-mutated MPN cells in ex vivo studies [46–48]. Givinostat is a novel and potent histone deacetylase inhibitor that in ex vivo experiments was shown to suppress JAK2-V617F as well as STAT3 and STAT5 protein activation, ultimately leading to the inhibition of *JAK2*-V617F-mutated cell proliferation without affecting *JAK2* wild-type cells [48]. Release of pro-inflammatory cytokines like IL-1, IL-6 and TNF-α by malignant cells is also shown to be inhibited with exposure to givinostat [49, 50]. A pilot phase 2 study of givinostat in patients with MPN (including 12 with PV) demonstrated significant reduction of splenomegaly and symptom burden, appreciable hematologic responses and a safe toxicity profile [51]. A subsequent phase 2 trial restricted to patients with PV unresponsive to hydroxyurea studied the addition of givinostat and reported impressive rate of pruritus resolution, good tolerance and a 50 and 55% complete (CR) and partial response (PR) rate, respectively [52]. More recently, the dose expansion phase of another phase 2 trial of givinostat monotherapy in 35 patients with previously-treated PV reported an 81% response rate, although grade ≤ 3 diarrhea, thrombocytopenia and creatinine elevations were observed in 35–50% of patients [35]. A phase 3 study comparing frontline givinostat to standard-of-care hydroxyurea in high-risk patients with PV is being planned [53] (Table 1). Similarly, IMG-7289 (bomedemstat) is a first-in-class, small molecule inhibitor of lysine-specific demethylase 1, which in in vitro and in vivo studies have shown to influence myeloid hematopoietic progenitor differentiation and self-renewal in *JAK2*-mutated cells [54]. A phase 2 study of IMG-7289 of patients with PV or ET refractory to prior cytoreductive therapy is underway (NCT04262141).

Another class of drugs being investigated for PV treatment is the MDM2 inhibitors. MDM2 acts a chief negative regulator of TP53, which is a pleiotropic and critical mediator of pro-apoptotic responses to cellular stress and damage [55]. MDM2-mediated TP53 regulation is multifactorial and includes direct inhibition of transcriptional activity as well as functioning as an E3 ubiquitin ligase modifier thus leading to TP53 proteasome-mediated degradation [55]. Overexpression of MDM2 downregulates TP53 activity in *JAK2*-mutated CD34+ MPN cells [56, 57]; conversely, in vitro treatment with an MDM2 inhibitor was shown to increase cellular TP53 levels/activity and consequently selective CD34+ PV cell death [56]. A phase 1 trial of idasanutlin, an oral MDM2 antagonist, in 13 patients with high-risk, previously-treated PV and ET (PV = 12, ET = 1) demonstrated a favorable toxicity profile with 7 out of 12 patients (58%) achieving CR or PR, including both symptomatic and molecular responses. The lone patient ET unfortunately developed deep venous thrombosis 1 week into therapy and was removed from the trial [58]. Interim analyses from an ongoing phase 2 study of idasanutlin for hydroxyurea-resistant/intolerant PV demonstrated that 9 of 16 (56%) patients achieved hematocrit control by 32 weeks of therapy [36]. Unfortunately, 63% of patients required dose modification with most prompted by significant nausea/vomiting and diarrhea [36] (Table 1). Recent data also suggest that idasanutlin stimulates the expansion of *TP53*-mutated clones, although these clones regressed with discontinuation of treatment [59]. Due to its side effect profile, the development of idasanutlin for PV management was discontinued. Novel and more potent MDM2 inhibitors have also been developed. KRT-232 was investigated in a phase 2 trial of patients with PV and hydroxyurea resistance/intolerance (NCT03669965), but the study was halted due to low accrual.

Telomerase activity inhibition via targeting of the RNA template of the telomerase subunit hTERC can inhibit the growth of megakaryocytic colony-forming units obtained from patients with ET while sparing the normal megakaryocytic proliferation of samples from healthy controls [60, 61]. Imetelstat, the telomerase activity inhibitor used in these preclinical studies, demonstrated a rapid, durable and impressive (89%) complete hematologic response rate in 18 patients with ET refractory to prior therapy [40] (Table 2).

The iron metabolism pathway has been the subject of recent and unique targeting for the management of PV, which is essentially treated by either inducing or exacerbating iron deficiency with serial therapeutic phlebotomies targeting a hematocrit goal of < 45% based on the CYTO-PV study results [62]; however, iron deficiency can produce significant symptomatology. Both iron deficiency and expanded erythropoiesis in patients with PV may lead to suppression of hepcidin, the chief regulator of iron homeostasis [63]. Lack of hepcidin enhances the availability of iron for erythropoiesis in patients with PV [37, 64]. PTG-300, a first-in-class, subcutaneous hepcidin mimetic, is in phase 2 testing for patients with low- and high-risk PV requiring therapeutic phlebotomies (NCT04057040). Interim data on 13 patients showed that the drug is well-tolerated and led to phlebotomy independence in all treated patients [37]. Additionally, PTG-300 normalized iron stores in as short as 4 weeks of therapy and improved patient’s symptom burden [37] (Table 1).

## Conclusion

Although most patients with PV or ET will enjoy a normal life expectancy, some higher-risk patients suffer complications and are at increased risk of disease transformation and early mortality. Endeavors aimed at improving the quality of life and outcomes of patients with PV and ET are focused on hematologic control and thrombotic risk reduction. Hydroxyurea or interferon therapy is the current standard of care for higher-risk patients with ruxolitinib or anagrelide being reserved for second-line management. Longer-acting interferons with more convenient administration schedules such as ropeginterferon alfa-2b, which appears to be disease-modifying and superior to hydroxyurea, offer more options for the patient with PV. Alternatively, combining current standard-of-care therapies may offer synergy and better patient outcomes, as demonstrated with the combination of ruxolitinib and PEG-IFN alfa-2a.

If available, clinical trials should be offered to patients who are failed by these therapies, which may soon be supplanted by agents with optimized JAK-STAT pathway inhibition. The novel agents exploiting mechanisms critical to MPN HSC proliferation, such as inhibitors of histone deacetylase, MDM2 and telomerase, are currently being studied. Hepcidin mimetics offer an opportunity to normalize the disrupted iron homeostasis observed in PV, while alleviating the need for therapeutic phlebotomies. Randomized trials of new agents in comparison to reference standards are necessary to confirm their value for patients with PV and ET. Correlative studies exploring molecular and metabolic changes may help to understand treatment potential to modify the course of disease. Continued high-impact research may soon foster the development of disease-modifying therapies for PV and ET and satisfy this need for the optimal management of patients with these MPNs.

## Data Availability

Not applicable.

## Abbreviations

alloHCT: Allogeneic hematopoietic stem cell transplantation
AML: Acute myeloid leukemia
CHR: Complete hematologic response
ET: Essential thrombocythemia
FDA: Food and Drug Administration
HDACi: Histone deacetylase inhibitor
HSC: Hematopoietic stem/progenitor cells
*JAK2*: *Janus kinase 2*
MDS: Myelodysplastic syndrome
MPN: Myeloproliferative neoplasm
ORR: Overall response rate
PEG-IFN alfa-2a: Pegylated interferon alfa 2a
PV: Polycythemia vera
